# Cognitive impairment, neuroimaging abnormalities, and their correlations in myotonic dystrophy: a comprehensive review

**DOI:** 10.3389/fncel.2024.1369332

**Published:** 2024-04-04

**Authors:** Yanyun Wu, Qianqian Wei, Junyu Lin, Huifang Shang, Ruwei Ou

**Affiliations:** Department of Neurology, West China Hospital, Sichuan University, Chengdu, China

**Keywords:** myotonic dystrophy, cognitive impairment, neuroimaging abnormalities, mechanism, comprehensive review

## Abstract

Myotonic dystrophy (DM) encompasses a spectrum of neuromuscular diseases characterized by myotonia, muscle weakness, and wasting. Recent research has led to the recognition of DM as a neurological disorder. Cognitive impairment is a central nervous system condition that has been observed in various forms of DM. Neuroimaging studies have increasingly linked DM to alterations in white matter (WM) integrity and highlighted the relationship between cognitive impairment and abnormalities in WM structure. This review aims to summarize investigations into cognitive impairment and brain abnormalities in individuals with DM and to elucidate the correlation between these factors and the potential underlying mechanisms contributing to these abnormalities.

## 1 Introduction

Myotonic dystrophy (DM) is a neuromuscular disease characterized by autosomal dominant inheritance, with a prevalence of 9.99 per 100,000 (Liao et al., 2022). DM is generally acknowledged as a condition that primarily affects skeletal muscle, leading to myotonia, progressive muscle weakness, and wasting (Vydra and Rayi, 2023). Additionally, it is known to cause abnormalities in multiple systems. Patients with DM may experience complications such as arrhythmia, cataracts, abnormal glucose tolerance, and gastrointestinal dysfunction (Rodbard et al., 2023).

DM is commonly categorized into two subtypes based on distinct genetic mutation sites: DM type 1 (DM1) and DM type 2 (DM2). DM1 and DM2 share numerous similarities but exhibit notable distinctions (Vydra and Rayi, 2023). DM1, also known as Steinert disease, is caused by an unstable CTG trinucleotide repeat expansion in the 3′ untranslated region (UTR) of the myotonic dystrophy protein kinase (*DMPK*) gene located on chromosome 19q13.3. This gene encodes *DMPK*, which is expressed in skeletal muscle and functions as a myosin kinase (Brook et al., 1992). DM2 results from an unstable tetranucleotide CCTG repeat expansion in intron 1 of the nucleic acid-binding protein (*CNBP*) gene (previously known as zinc finger 9 gene, *ZNF9*) on chromosome 3q21, consisting of a complex repeat motif (TG)n (TCTG)n (CCTG) n (Ranum et al., 1998). The expansion of the CCTG repeats, ranging from 75 to 10,000 times, leads to the manifestation of DM2 (Liquori et al., 2001).

DM1 can be classified into five distinct forms: congenital, childhood-onset, juvenile-onset, adult-onset (also known as classic form), and late-onset (Meola and Sansone, 2007). The congenital form is the most severe and is characterized by CTG extensions of more than 1,000 repeats. The resulting hypotonia and respiratory distress have a detrimental effect on both survival and development (Ho et al., 2015). Childhood-onset or juvenile-onset describes children who develop symptoms after the age of 1 year. Both of them develop symptoms similar to those of adults. The main difference between them is that childhood-onset is initially clinically apparent between the ages of 1 and 10 years, while juvenile-onset is apparent between 10 and 20 years (Stokes et al., 2019). The adult or classic form is the most common type of DM1. Additionally, the adult-onset form exhibits high variability. It can vary in presentation and severity among different individuals, even within the same family. The classic presentation is characterized by the presence of myotonia and muscle weakness (Joosten et al., 2023). The late-onset subtype, which typically manifests after the age of 40, is challenging to diagnose due to its subtle symptoms and is often associated with the development of cataracts (Yum et al., 2017).

The available evidence suggests the involvement of the central nervous system (CNS) in patients with DM. The recognition of cognitive impairment is increasingly gaining importance due to its significant impact on overall quality of life (Wenninger et al., 2018). However, the heterogeneity of clinical manifestations makes it challenging to identify specific features of cognitive impairment. Current cognitive assessments predominantly rely on scales like the Mini-Mental State Examination (MMSE), which may be influenced by subjective factors. Evidence has demonstrated that changes in imaging can provide insights into the location and extent of CNS involvement (Angelini and Pinzan, 2019). Moreover, numerous studies have established a correlation between cognitive impairment and neuroimaging abnormalities (Cabada et al., 2021; Labayru et al., 2022; Koscik et al., 2023). Finally, current research on mechanisms falls short of providing a comprehensive explanation for the observed abnormalities in the CNS and neuroimaging changes, as well as their interrelationship.

Therefore, this review investigates cognitive impairment and abnormalities in neuroimaging among patients with DM. It also explores their interrelationships to establish them as reliable markers of disease severity in cognitive impairment.

## 2 Cognitive impairment in DM

Ample evidence suggests cerebral involvement in DM (see Supplementary Table S1). Patients with DM may experience cognitive impairment, personality disorders, sleep disorders, fatigue, social difficulties, and other related issues, which are more pronounced in DM1 (Hamel, 2022). Among them, cognitive impairment is frequently observed in individuals with DM, expecially in those with DM1 (Table 1).

**Table 1:** Summary of literature on cognitive impairment in DM.

Author	Research type	DM subtype	DM	HC	Tools	Main findings
Rakocevic-Stojanovic (2014)	Cross-sectional	DM1	66	–	CANTAB, etc.	Cognitive impairment in DM patients could affect patients’ quality of life
Gaul (2006)	Cross-sectional	DM1 DM2	21 DM1 21 DM2	21	RCFT, CAL, VF, etc.	Frontotemporal functions were impaired in DM1 and DM2 patients, and declined over time
Modoni (2008)	Longitudinal	DM1	34	–	MMSE etc.	
Sansone (2007)	Longitudinal	DM1 DM2	56 DM1 29 DM2	–	MMSE, TMT, TLT, etc.	
Sistiaga (2010)	Cross-sectional	DM1	121	54	WAIS etc.	
Angeard (2011)	Cross-sectional	DM1	24	–	WAIS etc.	Both DM1 and DM2 patients had cognitive impairment, including executive function, visual–spatial construction abilities, memory, attention abilities, and other cognitive domains. The patients with DM2 were less severe than those with DM1. Intelligence impairment was common in DM, especially in CDM patients, and other domains like language and communication skills were also observed in patients with DM
Baldanzi (2016)	Cross-sectional	DM1	65	26	RVLT, ROCF, CBT etc.	
Fujino (2018)	Cross-sectional	DM1	60	–	MMSE, WAIS, VPTA, FAB, WCST, TMT etc.	
Peric (2017)	Cross-sectional	DM1 DM2	101 DM1 46 DM2	–	MMSE, ACE-R, WAIS, ST, RVLT, ROCF etc.	
Gallais (2017)	Longitudinal	DM1	115	–	WAIS etc.	
Peric (2022)	Cross-sectional	DM2	76	–	ACE-R, MMSE, etc.	
Zalonis (2010)	Cross-sectional	DM1	23	23	NPE	
Douniol (2012)	Cross-sectional	DM1	28	–	MINI, WISC, etc.	
Labayru (2019)	Longitudinal	DM1	75	54	CalCAP, PM47 etc.	
Sweere (2023)	Cross-sectional	DM1	45	–	NPE	
Kleberg (2014)	Cross-sectional	DM1	33	30	RBMT-E, VF, RVLT, RCFT etc.	Both DM1 and DM2 patients had cognitive impairment, including executive function, visual–spatial construction abilities, memory, attention abilities, and other cognitive domains. The patients with DM2 were less severe than those with DM1. Intelligence impairment was common in DM, especially in CDM patients, and other domains like language and communication skills were also observed in patients with DM
Woo (2019)	Cross-sectional	DM1	19	–	WAIS, ST, COWAT	
Rubinsztein (1997)	Cross-sectional	DM1	36		MMSE, WCST, etc.	
Ekstrom (2009)	Cross-sectional	DM1	55	–	WISC, VABS, etc.	
Filli (2020)	Cross-sectional	DM1	19	19	D-KEFS, RWT, TMT	
Kobayakawa (2012)	Cross-sectional	DM1	9	12	MMSE, FAB, ROCF	
Modoni (2004)	Cross-sectional	DM1	70	–	MMSE etc.	
Ricci (2022)	Longitudinal	DM1	10	–	Intellectual test, etc.	
Woodward (1982)	Cross-sectional	DM1	17	25	WAIS, WCST	
Steyaert (1997)	Cross-sectional	DM1	16	–	WISC, WAIS, etc.	
Romeo (2010)	Cross-sectional	DM1 DM2	50DM1 14DM2	44	PM47, ST, RCFT, etc.	
Fortin (2023)	Cross-sectional	DM1	30	–	CANTAB	
Tuikka (1993)	Longitudinal	DM1	35	–	WAIS etc.	Cognitive impairments differed in various forms of DM, and there was no severe cognitive decline over time
Bird (1983)	Cross-sectional	DM1	29	–	WAIS etc.	The severity of cognitive impairment could be affected by inheritance patterns, age at onset, disease duration, and CTG repeats
Huber (1989)	Cross-sectional	DM1	41	16	MMSE, VF, PM47, etc.	
Palmer (1994)	Cross-sectional	DM1	21	10	WAIS, ST, etc.	
Winblad (2016)	Longitudinal	DM1	37	–	RCFT, RVLT, TMT, ST	
Winblad (2006)	Cross-sectional	DM1	47	–	WAIS, RCFT, TMT, etc.	
Tremblay (2021)	Cross-sectional	DM1	11	–	WAIS, FAB, etc.	
Díaz-Leiva (2020)	Longitudinal	DM1	31	–	DST, BVRT, CPT, etc.	Neurocognitive progression in DM1 seemed to respond to a progressive pattern of degeneration Cognitive function declined both in patients with DM1 and DM2 over time
Fujino (2023)	Longitudinal	DM1	66	–	MMSE, WCST, SDMT	
Gliem (2019)	Longitudinal	DM1 DM2	16DM1 16DM2	17	BNT, TMT, etc.	
Labayru (2019)	Cross-sectional	DM1	31	57	ST, PM47, CalCAP, etc.	Neuropsychologic tests observed cognitive impairment and correlated with brain changes in DM1 and DM2 patients, including brain atrophy, WML, and hypoperfusion in specific brain regions
Meola (2003)	Cross-sectional	DM1 DM2	21DM1 19DM2	–	MMSE, TMT, ST, etc.	
Schneider-Gold (2015)	Cross-sectional	DM1 DM2	12DM1 16DM2	33	RVLT etc.	
Theodosiou (2022)	Cross-sectional	DM2	11	26	ECAS	
Langbehn (2021)	Cross-sectional	DM1	50	68	WAIS	
Caso (2014)	Cross-sectional	DM1	51	34	PM47, ACE-R, TMT, BNT, WAIS, WCST	
Baldanzi (2016)	Cross-sectional	DM1	30	30	RVLT, ROCF, CBT, TMT, ST, FAB, WCST	
Serra (2020)	Cross-sectional	DM1	31	26	social cognition, PM46	
Van (1995)	Cross-sectional	DM	26	25	WAIS, RVLT, WCST	No cognitive impairment was found in patients with early adult and adult-onset DM

CANTAB, Cambridge Neuropsychological Test Automated Battery; RCFT, Rey Complex Figure Test and Recognition Trial; CAL, Conditional-associative Learning; VF, Verbal Fluency; MMSE, Mini-Mental State Examination; TMT, Trail Making Test; TLT, Tower of London Test; WAIS, Wechsler Adult Intelligence Scale; RVLT, Rey Verbal Learning Test; ROCF, Rey-Osterrieth Complex Figure; CBT, Corsi’s Block Test; VPTA, Visual Perception Test for Agnosia; WCST, Wisconsin Card Sorting Test; FAB, Frontal Assessment Battery; ACE-R, Addenbrooke’s Cognitive Examination-Revised; ST, Stroop Test; NPE, neuropsychological examination; MINI, Mini-International Neuropsychiatric Interview; WISC, Wechsler Intelligence Scale for Children; CalCAP, California Computerized Assessment Package; PM47, Raven’s Progressive Matrices; RBMT-E, Rivermead Behavioral Memory Test-Extended; COWAT, Controlled Oral Word Association Test; VABS, Vineland Adaptive Behavior Scales; D-KEFS, Delis-Kaplan Executive Function System; RWT, Regensburg Word Fluency Test; DST, Digital Span Test; SDMT, Symbol Digit Modalities Test; BNT, Boston Naming Test; BVRT, Benton Visual Retention Test; CPT, Continuous Performance Test; ECAS, Edinburgh Cognitive and Behavioral Amyotrophic Lateral Sclerosis Screen.

A previous review summarized that cognitive impairment was observed in over 50% of DM1 patients, which manifests as a variable combination of global cognitive deficits across various domains, including social cognition, memory, executive function and visuospatial function (Rosado Bartolomé et al., 2022). It explicitly affects frontotemporal functions (Gaul et al., 2006; Sansone et al., 2007; Modoni et al., 2008; Sistiaga et al., 2010), the prominent characteristic of which is executive dysfunction (Baldanzi et al., 2016; Gallais et al., 2017; Peric et al., 2017; Fujino et al., 2018; Peric et al., 2022). This manifests as difficulties in planning, organizing, initiating tasks, and making decisions. Consequently, it can significantly impact the ability to effectively carry out daily activities, resulting in apathy and reduced physical activity. Additionally, visuospatial processing difficulties exist, encompassing the comprehension and interpretation of visual information about spatial connections among objects (Angeard et al., 2011; Douniol et al., 2012; Baldanzi et al., 2016; Gallais et al., 2017; Peric et al., 2017; Fujino et al., 2018; Labayru et al., 2020a; Peric et al., 2022; Sweere et al., 2023). Memory deficits, particularly in working memory and short-term memory domains, are prevalent among patients with DM1, leading to difficulties in recalling recent events and acquiring new knowledge (Gallais et al., 2017; Fujino et al., 2018; Woo et al., 2019; Sweere et al., 2023). Some patients with DM1 may also experience challenges in intelligence, which can impact their social interactions, language, and communication skills (Kobayakawa et al., 2012; Filli et al., 2020; Ricci et al., 2022; Fortin et al., 2023). It may manifest as difficulties with word retrieval, comprehension, and expression. These investigations enhance our understanding of the involvement of the central nervous system in DM1 and facilitate the formulation of corresponding therapeutic strategies.

The cognitive symptoms in DM1 patients exhibit significant variations due to the diverse onset patterns, encompassing a spectrum from mild to severe (Table 2). Congenital myotonic dystrophy (CDM) is a fetal disease that is characterized by CTG repeat extensions longer than 1,000 repeats. Typically, patients have an average intelligence quotient (IQ) below 70 and experience mental retardation, which may precede the development of muscle symptoms (Reardon et al., 1993; Spranger, 1997; Cascais et al., 2024). Apart from lower IQ, adaptive skills, executive dysfunction, sleep disorders, and autistic symptoms can be observed in CDM (Patel et al., 2024). These defects deeply affected the quality of life, even extending into adolescence for affected patients. It’s disheartening to know that despite multiple efforts, numerous patients are still unable to achieve a satisfactory life. Childhood-onset and juvenile-onset forms can display attention deficit hyperactivity disorder (ADHD) and autism spectrum disorder (ASD), along with difficulties in learning and other aspects, resulting in poor interpersonal relationships. Conversely, the adult-onset form can exhibit normal intelligence. As time passes, they may experience declines in various cognitive domains. The late-onset subtype, typically occurring after the age of 40, presents challenges in diagnosis due to its subtle symptoms and is frequently associated with the involvement of other systems. Cognitive impairment often occurs in numerous patients with late-onset DM1, although it is generally less severe compared to other forms.

**Table 2:** Differences between all forms of DM1.

	Congenital	Childhood-onset	Juvenile-onset	Adult-onset	Late-onset
CTG length	>1,000	50–1,000	50–1,000	50–1,000	50–100
Age at onset	From birth to 1 year	1–10	11–20	20–40	>40
Muscle system	Hypotonia, facial weakness, motor development delayed, reduced mobility	Myotonia, distal muscle weakness, and atrophy	Myotonia, distal muscle weakness, and atrophy	Distal muscle weakness, Myotonia	Mild myotonia, Muscle weakness
CNS system	Learning difficulties, ADHD, ASD, behavior problems, depression	Cognitive impairment, ADHD, ASD, sleep disorders, fatigue learning difficulties	Cognitive impairment, ADHD, ASD, sleep disorders, fatigue, learning difficulties	Cognitive impairment depression, fatigue, apathy, EDS, psychomotor delay, autistic features	Mild cognitive impairment, depression, fatigue, apathy, sleep disorders
Cognitive impairment	Mental retardation, behavioral abnormalities	Intelligence impairment, learning disabilities, attention, and visuospatial functions	Intelligence impairment, learning disabilities, attention, and visuospatial functions	Executive dysfunction, memory loss, visuospatial dysfunction, reduced attention span	Executive dysfunction, memory loss, visuospatial dysfunction, reduced attention span
Neuroimaging	Ventriculomegaly, macrocephaly, WML	WMHL, GM, and WM volume loss	WMHL, GM, and WM volume loss	WMHL, GM, and WM volume loss, atrophy	WMHL, GM, and WM volume loss, atrophy

ADHD, attention deficit hyperactivity disorder; ASD, autism spectrum disorder; EDS, excessive daytime sleep; WMHL, white matter hyperintense lesions; GM, grey matter; WM, white matter.

Multiple factors play an essential role in contributing to the variations in cognitive status among these forms, including the length of the CTG repeat expansion in the *DMPK* gene, age at onset, and inheritance pattern (Palmer et al., 1994; Winblad et al., 2006, 2016; Tremblay et al., 2021). An earlier onset and longer disease duration both indicate more severe cognitive deficits. Moreover, cognitive impairment tends to worsen progressively over time in adult-onset and late-onset forms, suggesting that there may be a positive correlation between cognitive decline and disease duration (Maresca et al., 2020; De Serres-Bérard et al., 2021). It provides evidence that brain changes in DM1 are more likely to be neurodegenerative (Labayru et al., 2019; Díaz-Leiva et al., 2020; Fujino et al., 2023).

The manifestation of DM2 is similar to DM1 in both the muscular and central nervous systems but less severe (Table 3) (Johnson et al., 2021). For example, almost all patients with DM1 experience cognitive impairment in multiple domains, while only one-third of DM2 patients may encounter cognitive difficulties (Sansone et al., 2007; Romeo et al., 2010; Gallais et al., 2017). Unfortunately, the amount of research on cognitive impairment in DM2 is not comparable to that conducted on DM1. Several studies suggest that patients with DM2 may exhibit better neuropsychological outcomes than those with DM1, implying potentially milder and less pervasive cognitive deficits (Peric et al., 2015; Gliem et al., 2019; Theodosiou et al., 2022). They indicate executive dysfunction associated with frontal and temporal lobe dysfunction, along with memory deficits and other cognitive challenges among individuals with DM2. However, it is mainly observed in older patients during testing or disease onset and those experiencing more pronounced muscle weakness (Peric et al., 2015).

**Table 3:** Comparisons for clinical features between DM1 and DM2.

		DM1	DM2
Prevalence		9.31/100,00	–
Pathogenic gene		*DMPK* gene	*CNBP* gene
Chromosome		19q13.3	3q21
Repeat expansion		(CTG)n	(CCTG)n
Expansion size		50–4,000	75–11,000
Form		Congenital, childhood-onset, juvenile-onset, adult-onset, late-onset	Adult-onset, late-onset
Muscular symptom		Distal limb, neck flexor, and bulbar weakness	Prominent proximal weakness and slight muscle wasting
Cognitive impairment	Execution	Yes	Yes
	Memory	Yes	Yes
	Visuospatial function	Yes	Yes
	Social cognition	Yes	Yes
	Attention	Yes	Yes
	Learning ability	Yes	No
	Intelligence	Yes	No
	Speech and language	Yes	No
Severity		Mild to severe	Mild to moderate
Associated factors		Inheritance pattern, age at onset, disease duration, repeat expansion	Age, age at onset, disease duration
Hypothesis		Neurodegeneration and neurodevelopment	Neurodegeneration
Brain involvement	Brain atrophy	Yes	Yes
	Dilatation of ventricles	Yes	Yes
	WMHLs	Yes	Yes
	Metabolism	Decreased	Decreased
	Blood perfusion	Hypoperfusion	Hypoperfusion
	Function connectivity	Decreased	Decreased

It is obvious that in various forms of DM, cognitive impairment becomes a significant problem, which increases the burden of life and healthcare. The exact reasons behind the diverse forms of cognitive impairment are not entirely understood. The different subtypes show varying disease progressions. Cognitive impairment is present from birth in CDM, while cognitive function in childhood, adult, and late-onset forms can develop normally but tends to decline over time. There is a need for a thorough investigation into cognitive impairment and its dynamic changes in different forms and subtypes of DM.

## 3 Neuroimaging abnormalities in DM

The cerebral involvement in DM1 and DM2 has been demostrated *in vivo* using diverse neuroimaging techniques (Table 4). Advancements in neuroradiological techniques have facilitated the potential for neuroimaging to identify DM (Minnerop et al., 2018). It entails employing various magnetic resonance imaging (MRI) techniques, such as traditional MRI to depict the basic brain structure and lesions, diffusion tensor imaging (DTI) to illustrate the microstructure in brain tissue, functional MRI (fMRI) to evaluate the metabolism, Fluorodeoxyglucose positron emission tomography (FDG-PET) to exam cerebral glucose metabolism, and single-photon emission computed tomography (SPECT) to evaluate cerebral perfusion. The neuroimaging features of DM exhibit variability and are not as pronounced as the clinical symptoms. These features include structural changes such as brain atrophy, ventricular enlargement, and white matter lesions (WML), as well as functional abnormalities such as hypoperfusion, decreased metabolism, and altered brain connectivity (see Supplementary Table S2).

**Table 4:** Summary of neuroimaging studies on DM.

Author	DM subtype	DM	Control	Tools	Main findings
Huber (1989)	Not specific	41	16	Structural MRI	1. Structural changes in neuroimaging were observed, including skull thickness, cerebral atrophy, ventriculomegaly, WML, WMHL, ATWML, HWMPST, and VRS were observed in cognitive impairment patients with DM 2. WML and brain atrophy were found in DM1 and DM2 patients but were more severely manifested in DM1 patients. ATWML was exclusively seen in DM1. Both were correlated to central nervous system involvement 1. Dilated convexity VRS might be one of the initial findings in MRI of DM, potentially contributing to the cognitive impairment of DM patients 2. The changes in brain imaging might be associated with the family history of lesions, disease duration, and inheritance pattern but not with the CTG repeat size
Abe (1994)	Not specific	14	14	Structural MRI	
Damian (1994)	Not specific	28	–	Structural MRI	
Hashimoto (1995)	Not specific	13	–	Structural MRI	
Bachmann (1996)	Not specific	40	–	Structural MRI	
Di Costanzo (2002)	DM1	66	–	Structural MRI	
Antonini (2004)	DM1	22	22	Structural MRI	
Magzhanov (2012)	DM1	20	10	Structural MRI	
Glantz (1988)	Not specific	14	12	Structural MRI	
Martinello (1999)	CDM	5	–	Structural MRI	
Di Costanzo (2002)	DM1	5CDM 20ADM	–	Structural MRI	
Hund (1997)	DM2	10	–	Structural MRI	
Di Costanzo (2001)	Not specific	20	20	Structural MRI	
Kuo (2005)	DM1	2CDM 4ADM	–	Structural MRI	
Censori (1994)	Not specific	25	25	Structural MRI	
Damian (1994)	Not specific	22	39 MS	Structural MRI	
Minnerop (2011)	DM1 and DM2	22	22	Structural MRI	
Bajram (2017)	DM1	13	–	Structural MRI	
Ogata (1998)	Not specific	12		Structural MRI	
Kornblum (2004)	DM1 and DM2	10DM1 9DM2	–	Structural MRI	
Leddy (2021)	DM1	29	15	Structural MRI	
Kassubek (2003)	DM1 and DM2	10DM1 9DM2	20	Structural MRI	
Di Costanzo (2001)	Not specific	41	41	Structural MRI	
Kuo (2008)	DM1	7	–	Structural MRI	
Sinforiani (1991)	Not specific	37	–	Structural MRI	
Savio (2011)	DM1	30	30	Structural MRI	
Di Costanzo (2008)	DM1	60	–	Structural MRI	
Meola (1999)	DM2	20	20	Structural MRI and H2O PET	The impaired visual–spatial function might be evident in DM2 and was strongly correlated with a reduction in regional cerebral blood flow observed in H_2_O PET brain scans
Cabada (2017)	DM1	42	42	DTI	Cerebral WM integrity and function were reduced in DM patients WM integrity and diffusivity were associated with cognitive impairment WM integrity loss was found in the genu, rostral body, anterior midbody, posterior midbody, and splenium in DM patients Tracking changes in WM integrity over time may be a valuable biomarker
Park (2018)	DM1	18	20	DTI	
Lopez-Titla (2021)	DM1	22	22	DTI	
Ota (2006)	Not specific	11	13	DTI	
Koscik (2021)	DM1	50	69	DTI	
Labayru (2022)	DM1	26	57	DTI	
Koscik (2023)	DM1	41	69	DTI	
Cabada (2021)	DM1	33	–	DTI	
Chang (1993)	Not specific	22	10	MRI and SPETC	The brain changes in MRI were affected by inheritance patterns.
Serra (2014)	DM1	27	16	fMRI	There was increased functional connectivity in the bilateral posterior cingulate and left parietal DMN nodes in DM1 patients
Vielhaber (2006)	DM1 and DM2	14DM1 15DM2	–	MRS	The concentration of N-acetyl aspartate was significantly reduced in both DM1 and DM2. In the DM1 patients, a concomitant depletion of creatine and choline levels was found, particularly in the frontal WM
Caliandro (2013)	DM1	29	30	fNIRS	DM1 patients exhibited prefrontal hypometabolism during a specific frontal cognitive task, and this hypometabolism in the prefrontal cortex was correlated with cognitive performance in DM1 patients
Fiorelli (1992)	Not specific	11	14	FDG/PET	Cortical glucose utilization rate was reduced in DM
Annane (1998)	Not specific	11	11	FDG/PET	The brain metabolism of glucose was impaired in DM patients
Laforce (2022)	DM1	12	2 AD	Tau PET	The Tau-PET tracer used in this study could not detect an *in vivo* Tau DM1 signature—however, most DM1 participants presented with elevated plasma NFL and GFAP levels

DM, myotonic dystrophy; DM1, myotonic dystrophy type 1; DM2, myotonic dystrophy type 2; CDM, congenital myotonic dystrophy; HC, healthy control; ADM, adult-onset myotonic dystrophy; mDM, DM with maternal inheritance; pMD, DM with paternal inheritance; fMRI, functional magnetic resonance imaging; DTI, diffusion tensor imaging; PET, positron emission tomography; SPECT, single photon emission computed tomography; MRS, magnetic resonance spectroscopy; fNIRS, functional near Infrared spectroscopy; ATWML, anterior temporal white matter lesions; WML, white matter lesion; WMHL, white matter hyperintensities lesions; HWMPST, hyperintensity of white matter at the posterior-superior trigone; WM, white matter; NFL, Neurofilament Light Chain; GFAP, Glial Fibrillary Acidic Protein.

Traditional structural MRI is the most frequently used neuroimaging technique to detect brain involvement in patients with DM1. Several studies have reported that patients with DM1 could suffer from widespread brain atrophy, resulting in a reduction in specific brain regions (Di Costanzo et al., 2002b; Antonini et al., 2004; Magzhanov et al., 2012). Shang et al. summarized that the reduction of GM1 volume involved the bilateral Rolandic operculum, bilateral posterior central gyrus, bilateral precentral gyrus, right insula, right Heschel gyrus, right superior temporal gyrus, bilateral supplementary motor area, bilateral middle cingulate gyrus/paracingulate gyrus, left paracentral lobule, and bilateral caudate nucleus (Jiang et al., 2022). Additionally, enlargement of the ventricles may occur due to grey matter (GM) and white matter (WM) volume reduction, a condition referred to as ventriculomegaly (Glantz et al., 1988; Martinello et al., 1999; Di Costanzo et al., 2002a). The findings of a study demonstrated the potential occurrence of multifocal cortical and thalamic atrophy in individuals with DM2 as well (Theodosiou et al., 2022).

The neuroimaging of patients with DM1 frequently reveals WM abnormalities. Conventional MRI in DM1 patients often detects WML or white matter hyperintensity lesions (WMHL) on T2-weighted imaging and fluid-attenuated inversion-recovery (FLAIR) pulse sequences (Di Costanzo et al., 2001a, 2002a; Kuo et al., 2005; Magzhanov et al., 2012), which may indicate a decrease in myelin sheaths. Studies have demonstrated that WMHL can be localized or widespread (Huber et al., 1989; Gliem et al., 2019; Garmendia et al., 2023). In patients with DM1, WMHL predominantly occur in the brain’s anterior temporal, frontal, parieto-occipital, and periventricular regions (Censori et al., 1994; Damian et al., 1994; Minnerop et al., 2011; Bajrami et al., 2017). Anterior temporal white matter lesions (ATWML) are more prevalent among patients with DM1 than those with DM2 (Ogata et al., 1998; Kornblum et al., 2004; Leddy et al., 2021). Although research on WML in DM2 may be limited compared to DM1, there are still reports indicating the presence of diffuse periventricular WMHL (Meola et al., 1999; Kassubek et al., 2003).

Over the past two decades, advanced MRI techniques, such as DTI, have been extensively utilized to investigate alterations in WM (Cabada et al., 2017; Park et al., 2018; Koscik et al., 2021; Lopez-Titla et al., 2021; Labayru et al., 2022). The use of DTI has also revealed alterations in WM microstructure among patients with DM1, indicating potential changes in the integrity of WM. Studies using region-of-interest (ROI) approach and tract-based spatial statistics (TBSS) have demonstrated a widespread decrease in fractional anisotropy (FA) and an increase in mean diffusivity (MD) in DM1 (Koscik et al., 2021; Laforce et al., 2022). It refers to the axons damage and myelin loss. Similarly, other investigations have consistently observed a symmetrical reduction in FA across various significant connections, projections, and commissural fibers (Wozniak et al., 2013; van Dorst et al., 2019). The proposed pattern of heightened impairment, predominantly affecting the fronto-temporo-parietal and subcortical regions, along with a loss of GM-related WM integrity, suggests that disruptions to neuronal networks may underlie the development of central nervous system symptoms in DM1 (De Antonio et al., 2016; van Dorst et al., 2019).

Dilated Virchow-Robin spaces (VRS) are reported to occur at a high frequency in patients with DM1 and may precede the development of WML (Bachmann et al., 1996; Di Costanzo et al., 2001b, 2002b). The presence of VRS is commonly observed in association with aging, vascular risk factors, neurodegenerative disorders, and neuroinflammatory diseases. However, the role of dilated VRS in DM1 remains inadequately understood.

Functional MRI has shown that there are more cerebral dysfunctions than just structural changes. Research findings have indicated that individuals with DM1 may exhibit cerebral hypoperfusion in perfusion MRI, which refers to a reduction in blood flow to the brain (Chang et al., 1993; Meola et al., 1999). Furthermore, patients with DM1 have been observed to display altered connectivity patterns in fMRI, particularly involving the default mode network (DMN), which is associated with self-referential thinking and internal mental processes (Serra et al., 2014; Okkersen et al., 2017; Minnerop et al., 2018; Serra et al., 2020). Additionally, decreased levels of N-acetyl aspartate (NAA) and hypometabolism were reported in patients with DM1 (Fiorelli et al., 1992; Annane et al., 1998; Vielhaber et al., 2006; Caliandro et al., 2013). At the same time, there is a lack of comprehensive research on functional MRI.

Nevertheless, these modifications may present distinctly across different subcategories of individuals with DM1. Evidence supports that ventriculomegaly or macrocephaly regularly occurs in CDM (Garcia-Alix et al., 1991). Neuroimaging studies in these patients reveal that more than 50% of them exhibit ventricular dilatation, which is present at birth. This finding supports the hypothesis of a prenatal origin for mental retardation (Meola and Sansone, 2007). A thorough review has made a comprehensive summary of them (Angelini and Pinzan, 2019). Diffuse microstructural WM damage with widespread FA reduction and increased mean diffusivity in fibers in noncongenital patients with DM1 associated with GM volume reduction were reported in numerous studies (Wozniak et al., 2013). The magnetic resonance spectroscopy (MRS) investigation revealed decreased NAA concentrations in both the grey and WM regions of DM1 patients, specifically those affected by congenital and juvenile forms, providing evidence of potential disruptions in neuronal maturation within the brain (Evangelisti et al., 2022).

In summary, various neuroimaging techniques, including the identification of both traditional structural modifications and functional changes, indicate that distinct mechanisms play a role in brain alterations in DM1 patients and suggest the presence of microstructural abnormalities.

The presentation of DM2 exhibits similarities to DM1 in both the muscular and central nervous systems, albeit with a milder manifestation. In DM2 patients, conventional brain MRI findings may appear within normal limits. However, diffuse WM changes can be observed in advanced stages or more severe cases, although they may exhibit less pronounced characteristics or differ from those seen in DM1. Surprisingly, a limited number of studies have reported that a subset of DM2 patients with diffuse and confluent WMHL similar to those found in cerebral autosomal dominant arteriopathy with subcortical infarcts and leukoencephalopathy (CADASIL) (Renard and Menjot De Champfleur, 2018).

Evidence also indicates a correlation between various factors and the abnormalities observed in brain imaging. Muscular and genetic features are associated with brain abnormalities in DM1, as evidenced by the changes in different forms (Labayru et al., 2019). Individuals with extended CTG repeats, prolonged disease duration, and specific inheritance patterns are more likely to experiencing sifnificant structural and functional changes in DM1 (Di Costanzo et al., 2001a, 2002a, 2008).

A 10-year longitudinal study reported a comparable decline in GM and WM volume loss and an increase in WML among individuals with childhood-onset, adult-onset, and late-onset DM1 (Labayru et al., 2020b). Furthermore, it was discovered that new GM and WM areas were impacted by disease progression in both childhood and adult-onset DM1 patients, in addition to the initially affected regions. The childhood-onset presented a reduction in both GM and WM volumes at baseline and during follow-up. The longitudinal analyses revealed that only adult and late-onset patients experienced a significant decline in WM volume over time (Garmendia et al., 2023). Other longitudinal studies have provided novel insights into the prevalence of WML in adult-onset DM1 and the progression of brain atrophy over time (Cabada et al., 2021; Koscik et al., 2021). However, a recent 5-year longitudinal study revealed no significant progression in the changes of GM and WM in patients with DM1 and DM2, suggesting either a slowly progressive process or a stable course of cerebral changes in middle-aged and adult-onset patients (Gliem et al., 2019). Microstructural WM changes might be attributed to development changes in congenital or child-onset forms, while GM degeneration may be more closely associated with a degenerative process related to aging. Additionally, WM abnormalities in the juvenile form may contribute to GM degeneration. Moreover, research using magnetization transfer imaging on DM indicated reduced magnetization transfer (MT) ratios in WMHL compared to normal-appearing white matter (NAWM), showing that structural changes in the WM may progress during the clinical course of DM. The independence of WM and GM neurodegenerative processes is suggested by the presence of WM T2 hyperintense lesions, and no correlation with GM loss is observed (Naka et al., 2002). It is generally suggested that both developmental and neurodegenerative changes are observed in DM. Cognitive impairment in DM is more likely to be neurodegenerative due to natural aging processes, despite differing results.

## 4 Correlation between cognitive impairment and brain changes

Extensive research has been conducted to better understand the cerebral changes associated with cognitive impairment in DM (Labayru et al., 2019).

Numerous studies have consistently reported that cognitive decline among patients with DM is closely associated with the degree of brain involvement, which can be found in neuroimaging (Cabada et al., 2021; Koscik et al., 2021; Lopez-Titla et al., 2021; Labayru et al., 2022). Specific brain regions, such as the anterior temporal lobes and basal ganglia, were significantly affected in social cognitive dysfunction. Atrophy and microstructural damage were found in DM1 patients in these regions. Bilateral ATWML may be a contributing factor to intellectual impairment in DM1, as it was observed in these patients. According to previous pathological findings, the loss and disordered arrangement of myelin sheaths and axons, along with heterotopic neurons raised the possibility that the ATWML is compatible with focal dysplasia of WM (Ogata et al., 1998). A negative correlation was observed between hippocampal volume and the Perceptual Reasoning Index (PRI) as well as Processing Speed Index (PSI) scores in patients with DM1 (Langbehn et al., 2021). Individuals with WM damages experienced more severe cognitive impairments, especially those with a higher burden of WMHL. This was associated with impairments in memory, executive function, reasoning, and visuospatial abilities (Bajrami et al., 2017).

Multiple studies have investigated the associations between cognitive performance and functional brain changes in patients with DM1 (Sinforiani et al., 1991; Bajrami et al., 2017; Lopez-Titla et al., 2021; Labayru et al., 2022). A DTI study in childhood-onset DM1 revealed abnormal WM integrity, demonstrating a strong correlation between FA, intelligence, and executive functioning (Koscik et al., 2023). Another analysis focusing on specific ROI in patients with DM1 found a significant association between FA and MD, particularly impacting working memory in the frontal and temporal lobes (Wozniak et al., 2013). Additionally, a significant correlation was observed between the decline in FA in the frontal, temporomedial, and parietal lobes and specific deficits in memory impairments (Lopez-Titla et al., 2021). Furthermore, studies using FDG-PET in DM1 have demonstrated an association between hypometabolism in brain lobes and executive dysfunction as well as attention deficit (Weber et al., 2010; Weijs et al., 2021; Sweere et al., 2023).

Longitudinal studies that track these changes over time have provided valuable insights into the progressive impact on cognitive function and WM. The degree of WM involvement is associated with declining cognitive function (Sansone et al., 2007). A longitudinal study found that the Rey-Osterrieth Complex Figure (ROCF) and two California Computerized Assessment Package (CALCAP) measures were positively correlated with the percentage of WM volume loss in adult and late-onset DM1 (Labayru et al., 2020b). Lower scores on neuropsychological tests were associated with higher percentage of total WM volume loss. Another longitudinal study using DTI in DM1 patients revealed a significant deterioration in WM integrity over time compared to healthy controls (Labayru et al., 2022). The FA values exhibited positive correlations with the visuo-construction domain and intellectual functioning. However, another longitudinal study indicated that the functional performance of middle-aged adult-onset patients improved at a slower rate (Gliem et al., 2019). In conjunction with a Bayesian stacked random forest model, a diffusion-based model using WM fiber tracts as predictors was employed to diagnose and predict outcomes in DM (Kamali et al., 2021). This work highlights the potential of neuroimaging in comprehending and monitoring clinical performance, especially cognitive impairment.

## 5 Potential mechanisms in cognitive impairment and brain changes

Although genetically distinct, DM1 and DM2 share a common pathogenic mechanism characterized by the formation of nuclear inclusions, sequestration of regulatory proteins, defects in alternative splicing, and repeat-associated non-ATG (RAN) translation (Liu et al., 2021).

A toxic RNA-mediated mechanism has been proposed based on the detection of abnormal RNA aggregates in muscle neurons obtained from post-mortem biopsies of patients with DM (Wheeler and Thornton, 2007). The aberrant RNAs are widely expressed in cortical and subcortical neurons, forming imperfect double-stranded hairpin structures that result in the deregulation of RNA-binding proteins (RBPs) responsible for mRNA splicing, such as muscleblind-like (MBNL) proteins and CUG RNA-binding protein (CUGBP1) (Giménez-Bejarano et al., 2023). The accumulation of these aberrant RNAs in the neuronal nucleus compromises a specific developmental program of alternative splicing (Lin et al., 2006; López-Martínez et al., 2020; Soltanzadeh, 2022). The dysregulation leads to a range of symptoms in patients with DM1. The RNA gain-of-function mechanism involves the accumulation of expanded CUG or CCUG transcripts within the cell nuclei, leading to the formation of ribonuclear inclusions.

The MBNL proteins play a prominent role in DM pathogenesis, as evidenced by the sequestration of each of the three MBNL isoforms (MBNL1, MBNL2, and MBNL3) by CUG RNAs in the cell nuclei (Meola and Cardani, 2015). The expression of MBNL1 is most prominent in the heart and muscles, indicating its predominant role in regulating alternative splicing. The expression of MBNL2 is prominently observed in the brain, while its levels decrease in muscle during postnatal development. It serves a related function in the central nervous system. Conversely, MBNL3 is expressed in the placenta and muscle satellite cells, although its functions *in vivo* remain unknown. Both MBNL1 and MBNL2 play essential roles in neuronal maturation. The MBNL1 is crucial for promoting the growth of neuronal dendrites, whereas MBNL2 is involved in overseeing developmental transitions related to alternative splicing and polyadenylation. Dysfunction of MBNL1 and MBNL2 may result in disrupted neuronal transmission and cognitive behavior, underscoring their significance in modulating synaptic transmission. There used to be MBNL2 knock-out rodent models that exhibited cognitive impairment and depressive-like behavior (Wang et al., 2018; Ramon-Duaso et al., 2019). An increased nuclear level of calpain-2 and a reduced MBNL2 level were observed in DM1 (Wang et al., 2022). Moreover, inhibiting the nuclear translocation of calpain-2 effectively prevented the degradation of MBNL2 and preserved its function in regulating RNA processing in the adult-onset form. Goodwin et al. found that MBNL2 protein also exhibits binding affinity for the CCUG RNA repeats in DM2 brain tissue (Goodwin et al., 2015). Therefore, MBNL2 might play an essential role in the CNS involvement of patients with DM.

Moreover, the discovery of repeat-associated non-ATG (RAN) translation and its increasing correlation with neurodegenerative disorders resulting from microsatellite expansions indicates that RAN proteins play a pivotal role in the neurological manifestations of DM (Zu et al., 2011). The study demonstrated that the CCTG expansion in DM2 expresses sense and antisense tetrapeptide poly-(LPAC) and poly-(QAGR) RAN proteins, which are modulated by MBNL through nuclear sequestration and are also found in neurons and accumulate within WM (Zu et al., 2017). However, the connection between the toxic RNA hypothesis and CNS involvement remains incomplete.

The RNA-seq analysis of both GM and WM tissue samples from the brains of patients with DM1 revealed a potential correlation between mis-spliced events and cerebral changes. Surprisingly, these events dominantly occurred in astrocytes and oligodendrocytes, which might be related to substantial WM damage (Yoshizumi et al., 2023). Another study found that the splicing abnormalities tended to be more prominent in the WM glial cells than in the GM neurons (Nakamori et al., 2022). Masayuki et al. used a particular approach to detect the neuronal cells in the cortex and glial cells in the WM (Nakamori et al., 2022). They found that cortical neurons had more unstable repeats and higher methylation compared to WM glial cells. These findings indicate that GM and WM are involved in patients with DM.

A study investigated the association between *DMPK* blood DNA methylation (DNAm) and cognitive functions in DM1 patients, revealing its potential role in predicting future cognitive decline at baseline (Breton et al., 2020). However, no methylation was observed in the *CNBP* gene of DM2 patients, neither in their blood cells nor muscles (Santoro et al., 2018). Furthermore, there is no evidence indicating methylation occurring in brain tissue among both DM1 and DM2 patients. The discussion about its association with neuroimaging changes was omitted.

According to the most recent systematic review, the primary neuropathological findings include WM changes, cellular changes, and deposition of protein and nucleotide (Weijs et al., 2021). In deep and subcortical WM, there was myelin loss with varying axonal loss, dilation of perivascular spaces, gliosis, and capillary hyalinization. Additionally, widened perivascular spaces were consistent with the observed features on MRI scans. One study proposed a hypothesis suggesting that the increased burden caused by microvascular changes, inadequate drainage of interstitial fluid, and degradation of protein products may serve as mechanisms for anterior temporal WML in DM1 (Renard and Menjot De Champfleur, 2018). However, it is worth noting that these changes may not be observed in all individuals with DM. It is essential to distinguish between DM and CADASIL, as both conditions can present with anterior temporal lobe hyperintensities and WMHL (Liu et al., 2022). Moreover, this suggests that the occurrence of these changes depends on the disconnection of the cortical regions due to the breakdown of the WM tract (Simoncini et al., 2020). A further study has demonstrated that cerebrospinal fluid (CSF) and blood neurofilament light chain (NFL) may serve as biomarkers of CNS involvement (Rossi and Silvestri, 2023). Neuronal loss was not observed in patients with CMD, but it was demonstrated in a subset of patients with adult-onset. However, these results were inconclusive. Several studies have shown that it may be related to clinical features of excessive daytime sleepiness and ventilatory dysfunction (Ono et al., 1996; Zalonis et al., 2010). Neurofibrillary tangles (NFTs), consisting of mis-spliced tau protein, were observed in the entorhinal to transentorhinal cortices in all patients with DM1 and exhibited a generally widespread distribution (Vermersch et al., 1996; Maurage et al., 2005; Iwasaki, 2018). However, limited neuropathological data is available for DM2; similar brain tau pathology has also been found in DM2 cases (Maurage et al., 2005). Nonetheless, no evidence of amyloid plaques associated with DM was found. Whether the cognitive impairment reported in DM is related to the development of tau pathology remains unclear. In a review of the pathogenesis and pathology of CNS deficits in DM1, the authors summarized the possible causes that may contribute to CNS damage (Liu et al., 2021).

The evidence strongly suggests that the brain manifests various pathologies that significantly impact its function, mainly cognitive function. However, further investigation is required to elucidate the underlying mechanisms in DM.

## 6 Conclusion

This review provides a comprehensive overview of cognitive impairment and brain changes in patients with DM, emphasizing the importance of neuroimaging for early detection and management. Future prospective cohort studies with larger sample sizes are warranted to elucidate its progression. At the same time, further investigation into underlying mechanisms is crucial for developing targeted therapeutic interventions that can improve patient outcomes.

## Author contributions

YW: Conceptualization, Writing – review & editing, Writing – original draft, Data curation, Investigation, Visualization, Methodology. QW: Conceptualization, Supervision, Validation, Writing – original draft, Writing – review & editing, Data curation. JL: Data curation, Investigation, Supervision, Validation, Writing – original draft, Writing – review & editing. HS: Conceptualization, Supervision, Writing – review & editing. RO: Conceptualization, Investigation, Writing – original draft, Writing – review & editing, Supervision, Methodology.
