# Supplementary material for: Cognitive impairment, neuroimaging abnormalities, and their correlations in myotonic dystrophy: a comprehensive review
Source: Front Cell Neurosci. 2024 Apr 4;18:1369332. doi: 10.3389/fncel.2024.1369332 (PMC11024338; doi:10.3389/fncel.2024.1369332)
Supplement: Supplementary file 1 [file Data_Sheet_1.DOCX]

**Table S1**. Summary of literature on cognitive impairment in DM.

| Author | Research Type | MD Subtype | MD | HC | Tools | Main Findings |
| --- | --- | --- | --- | --- | --- | --- |
| Rakocevic-Stojanovic (2014) (1) | cross-sectional | DM1 | 66 | / | CANTAB, etc. | 1. There are discrepancies between verbal and visual memory; 2. Visuospatial deficits in DM1 may reflect a fronto-parietal network involvement; 3. Executive abilities and processing speed deficits were less severe than mental flexibility, inhibition, or working memory in DM1. |
| Gaul (2006) (2) | cross-sectional | DM1  DM2 | 21 DM1 21 DM2 | 21 | RCFT, CAL, VF, etc. | 1. Global atrophy correlated with executive and visuo-spatial abilities; 2. TBSS revealed associations between DTI indexes and cognitive performances; 3. Disrupted complex neuronal networks can underlie cognitive impairment in DM1. |
| Modoni (2008) (3) | longitudinal | DM1 | 34 | / | MMSE etc. | Several cognitive functions were affected in patients with DM1, including executive and amnesic domains with visuo-spatial involvement. |
| Sansone (2007) (4) | longitudinal | DM1  DM2 | 56 DM1 29 DM2 | / | MMSE, TMT, TLT, etc. | 1. Females exhibit a tendency towards poorer cognitive function than males; 2. Limited cognitive ability correlated with the maternal inheritance of the gene and results in severe physical handicap, but there were occasional individual variations; 3. Strongest affected cognitive abilities were verbal and informational, whereas the weakest ones were immediate recall, abstraction and spatial manipulation and orientation. |
| Sistiaga (2010) (5) | cross-sectional | DM1 | 121 | 54 | WAIS etc. | WM damage, through a disconnection between GM structures, is likely to be the major contributor to cognitive impairment in DM1. |
| Angeard (2011) (6) | cross-sectional | DM1 | 24 | / | WAIS etc. | Neurocognitive progression in DM1 seems to respond to a progressive pattern of degeneration. |
| Baldanzi (2016) (7) | cross-sectional | DM1 | 65 | 26 | RVLT, ROCF, CBT etc. | Childhood-onset DM1 patients have borderline low intelligence and frequent impairments in attention and visual–spatial construction abilities. |
| Fujino (2018) (8) | cross-sectional | DM1 | 60 | / | MMSE, WAIS, VPTA, FAB, WCST, TMT etc. | Children and adolescents with DM1 exhibit significant cognitive and adaptive problems. |
| Peric (2017) (9) | cross-sectional | DM1  DM2 | 101 DM1  46 DM2 | / | MMSE, ACE-R, WAIS, ST, RVLT, ROCF etc. | 1. Cognitive impairments in patients with DM1 are attenuated within the attentional domains; 2. Cognitive processes play a crucial role in the maintenance of walking function in DM1. |
| Gallais (2017) (10) | longitudinal | DM1 | 115 | / | WAIS CANTAB etc. | 1.The Ruff 2 and 7 and Stroop Color-Word tests are two reliable paper-pencil measures. The Multitasking test shows the best reliability of the selected CANTAB tests.  2.Paper-pencil tests are more reliable than computerized tests in DM1. |
| Peric (2022) (11) | cross-sectional | DM2 | 76 | / | ACE-R, MMSE, etc. | DM1 patients exhibited cognitive impairment in attention/working memory, executive function, processing speed, and visuo-constructive ability, and they can be potential contributors to reduced QoL. |
| Zalonis (2010) (12) | cross-sectional | DM1 | 23 | 23 | NPE | Cognitive function declined in patients with DM1 overtime. |
| Douniol (2012) (13) | cross-sectional | DM1 | 28 | / | MINI, WISC, etc. | 1. A significant worsening in cognition over time was observed;  2. The progression in cognitive scores correlated with age and disease duration, but not with nCTG, muscular impairment, or education at baseline; 3. The rate of decline was higher among the late-onset phenotype than in the adult phenotype; 4. Executive functions, language, and visual memory are impaired earlier in adult life, while verbal memory, visual attention, and processing speed decline later. |
| Labayru (2019) (14) | longitudinal | DM1 | 75 | 54 | CalCAP, PM47 etc. | 1. No significant disease-related progression of gray and white matter involvement is observed over a period of five years in patients with DM1 and DM2 patients; 2. Minor neuropsychological progression was observed in DM1 and DM2. |
| Sweere (2023) (15) | cross-sectional | DM1 | 45 | / | NPE | Compared to controls, both patients with DM1 and DM2 were inferior in tests of prefrontal functioning. |
| Kleberg (2014) (16) | cross-sectional | DM1 | 33 | 30 | RBMT-E, VF, RVLT, RCFT etc. | DM1 patients with severe intellectual disturbance are more likely to inherit the disease from their mother, and are related to focal white matter lesions. |
| Woo (2019) (17) | cross-sectional | DM1 | 19 | / | WAIS, ST, COWAT | DM1 patients have deficits in facial memory ability, which was associated with reduced construction- and visual memory ability. |
| Rubinsztein  (1997) (18) | cross-sectional | DM1 | 36 |  | MMSE, WCST, etc. | Adult-onset DM1 is associated with the theory of mind dysfunction, which could be due to the brain lesions associated with this disease. |
| Ekstrom (2009) (19) | cross-sectional | DM1 | 55 | / | WISC, VABS, etc. | 1. Age poses vulnerability to grey matter loss in specific areas in DM1; 2. White matter alterations in DM1 may be developmental; 3. Muscular and genetic features are associated with brain abnormalities in DM1; 4. Neuropsychology is an unspecific but strong predictor of gray matter damage in DM1. |
| Filli (2020) (20) | cross-sectional | DM1 | 19 | 19 | D-KEFS, RWT, TMT | A domain-specific progressive cognitive decline is observed in DM1, with visuospatial/visuoconstructive abilities showing the greatest vulnerability to the passage of time. |
| Kobayakawa  (2012) (21) | cross-sectional | DM1 | 9 | 12 | MMSE, FAB, ROCF | Enlarged hippocampal volume may contribute to cognitive impairment in adult-onset DM1. |
| Modoni (2004) (22) | cross-sectional | DM1 | 70 | / | MMSE etc. | There is a specific cognitive and behavioral profile in DM2 and DM1, and this profile is associated with hypoperfusion in frontal and parieto-occipital regions of the brain. |
| Ricci (2022) (23) | longitudinal | DM1 | 10 | / | Intellectual test etc. | CDM exist in mental retardation, and adults experience an aging-related decline in frontal and temporal cognitive functions. |
| Woodward (1982) (24) | cross-sectional | DM1 | 17 | 25 | WAIS, WCST | Cognitive impairment is confined to frontotemporal functions in adult DM1 patients, with a tendency towards a decline with increasing aging. |
| Steyaert (1997) (25) | cross-sectional | DM1 | 16 | / | WISC, WAIS etc. | Inheritance pattern is one important moderating variable in determining the impact of the DM gene on cognitive functioning. |
| Romeo (2010) (26) | cross-sectional | DM1  DM2 | 50DM1 14DM2 | 44 | PM47, ST, RCFT, etc. | 1. Virtually all DM1 patients have cognitive defects with approximately 2–3 cognitive domains affected. One-third of DM2 patients have completely normal; neuropsychological findings, and in the other two-thirds approximately 1–2 domains are affected; 2. Visuospatial and executive dysfunctions seem to be the main cognitive defects. |
| Fortin (2023) (27) | cross-sectional | DM1 | 30 | / | CANTAB | 1.The most affected cognitive domains in DM2 are visuospatial, executive, and naming; 2.ACE-R is more sensitive than MMSE to detect cognitive deficit in DM2. |
| Tuikka (1993) (28) | longitudinal | DM1 | 35 | / | WAIS etc. | 1. Patients with juvenile form of DM1 have a lower QoL than adult-onset DM1 patients; 2. Significant predictors of QoL in patients with juvenile-onset DM1 were fatigue and visuospatial abilities; 3. Significant predictors of quality of life in patients with adult-onset DM1 were fatigue and level of education; 4. Clear influence of different central manifestations on QoL was found in patients with DM1. |
| Bird (1983) (29) | cross-sectional | DM1 | 29 | / | WAIS etc. | Congenital and childhood-onset patients both have intellectual disability. A reduction of developmental/intelligence quotient after 2 years is observed in re-tested patients. |
| Huber (1989) (30) | cross-sectional | DM1 | 41 | 16 | MMSE, VF, PM47, etc. | 1. A more severe involvement of the brain is observed in DM1 compared to DM2; 2. A temporo-insular diffuse lesional pattern, specific for DM1, is observed in MRI; 3. Greater expansion size is a risk factor for more extensive brain involvement in DM1. |
| Palmer (1994) (31) | cross-sectional | DM1 | 21 | 10 | WAIS, ST, etc. | Memory function may be more sensitive to small CTG repeat expansions than general intelligence |
| Winblad (2016) (32) | longitudinal | DM1 | 37 | / | RCFT, RVLT, TMT, ST | In both DM1 and DM2 frontal cognitive impairment (attentional) worsens over time but does not extend to additional areas of cognition, but DM2 was less severe than DM1 |
| Winblad (2006) (33) | cross-sectional | DM1 | 47 | / | WAIS, RCFT, TMT etc. | 1. GM and WM atrophy is observed in DM1 and DM2; 2. Specific functional-structural associations exist between morphological changes and cognitive impairment, depression, and daytime sleepiness. |
| Tremblay (2021) (34) | cross-sectional | DM1 | 11 | / | WAIS, FAB etc. | A prominent deficit of decision-making in patients with DM1. It might be related to increased connectivity between ventral tegmental area and brain areas critically involved in the reward/punishment system and social cognition. |
| Díaz-Leiva (2020) (35) | longitudinal | DM1 | 31 | / | DST, BVRT, CPT, etc. | The involvement of the CNS in adult DM1 patients is significantly influenced by CTG repeats, while cognitive impairment predominantly affects the fronto-parietal lobe. |
| Fujino (2023) (36) | longitudinal | DM1 | 66 | / | MMSE, WCST, SDMT | Patients with DM1 have an intelligence level below the population mean, and ADHD is most frequent psychiatric diagnosis. |
| Gliem (2019) (37) | longitudinal | DM1  DM2 | 16DM1 16DM2 | 17 | BNT, TMT, etc. | Children with DM1 may have specific cognitive deficits, most frequently affecting working memory, attention, and visuospatial functions, in addition to the previously described global intellectual impairments. |
| Labayru (2019) (38) | cross-sectional | DM1 | 31 | 57 | ST, PM47, CalCAP etc. | DM2 may be associated with multifocal cortical and thalamic atrophy, which is likely to underpin the range of cognitive manifestations mostly characterized by executive impairment and specifically by impaired social cognition. |
| Meola (2003) (39) | cross-sectional | DM1  DM2 | 21DM1 19DM2 | / | MMSE, TMT, ST, etc. | Adults with the childhood phenotype of DM1 demonstrate a relative dependence on others in terms of money management and home management & transportation, indicating a level of dependence that is associated with cognitive impairments. |
| Schneider-Gold (2015) (40) | cross-sectional | DM1  DM2 | 12DM1 16DM2 | 33 | RVLT etc. | 1. Cognitive function in adult-onset patients with DM1 is normal, and congenital form with moderate or mild mental retardation; 2. In follow-up, there was no severe cognitive impairment over time. |
| Theodosiou  (2022) (41) | cross-sectional | DM2 | 11 | 26 | ECAS | Hardly any evidence of cognitive impairment was found in DM patients with early adult and adult onset. |
| Langbehn (2021) (42) | cross-sectional | DM1 | 50 | 68 | WAIS | Compared to normal controls, DM1 patients have deficits in several cognitive abilities and are associated with CTG repeat expansion size in blood which can use as a broad predictor. |
| Caso (2014) (43) | cross-sectional | DM1 | 51 | 34 | PM47, ACE-R, TMT, BNT, WAIS, WCST | Both earlier onset and longer duration of the disease in DM1 patients are indicative of more cognitive deficits. |
| Baldanzi (2016) (44) | cross-sectional | DM1 | 30 | 30 | RVLT, ROCF, CBT, TMT, ST, FAB, WCST | Verbal memory impairment significantly deteriorated in juvenile-onset DM1 patients when compared to the adults-onset DM1 patients. |
| Serra (2020) (45) | cross-sectional | DM1 | 31 | 26 | social cognition, PM46 | 1. WAIS score is significantly lower in DM than in HC; 2. The motor symptoms and mental impairment were not correlated, suggesting that different clinical features of this systemic disease are variably penetrant. |
| Van (1995) (46) | cross-sectional | DM | 26 | 25 | WAIS, RVLT, WCST | Executive function, complex attention, memory, constructions, and reasoning were slightly affected which is mostly related to the dysfunction of the frontal association cortex and its underlying neural networks. |

Note: CANTAB, Cambridge Neuropsychological Test Automated Battery; RCFT, Rey Complex Figure Test and Recognition Trial; CAL, Conditional-associative Learning; VF, Verbal Fluency; MMSE, Mini-Mental State Examination; TMT, Trail Making Test; TLT, Tower of London Test; WAIS, Wechsler Adult Intelligence Scale; RVLT, Rey Verbal Learning Test; ROCF, Rey-Osterrieth Complex Figure; CBT, Corsi's Block Test; VPTA, Visual Perception Test for Agnosia; WCST, Wisconsin Card Sorting Test; FAB, Frontal Assessment Battery; ACE-R, Addenbrooke's Cognitive Examination-Revised; ST, Stroop Test; NPE, neuropsychological examination; MINI, Mini-International Neuropsychiatric Interview; WISC, Wechsler Intelligence Scale for Children; CalCAP, California Computerized Assessment Package; PM47, Raven’s Progressive Matrices; RBMT-E, Rivermead Behavioral Memory Test-Extended; COWAT, Controlled Oral Word Association Test; VABS, Vineland Adaptive Behavior Scales; D-KEFS, Delis-Kaplan Executive Function System; RWT, Regensburg Word Fluency Test; DST, Digital Span Test; SDMT, Symbol Digit Modalities Test; BNT, Boston Naming Test; BVRT, Benton Visual Retention Test; CPT, Continuous Performance Test; ECAS, Edinburgh Cognitive and Behavioral Amyotrophic Lateral Sclerosis Screen; QoL, quality of life; ADHD, attention deficit hyperactivity disorder .

**Table S2**. **Summary of neuroimaging studies on DM.**

| Author | DM subtype | DM Sample | Controls | Investigation | Main Findings |
| --- | --- | --- | --- | --- | --- |
| Glantz  1998(47) | Not specific | 14 | 12 | structural MRI | Patients with DM exhibited a higher prevalence of ventriculomegaly and a distinct lumpy and/or thick pattern of periventricular hyperintensity when compared to age-matched controls. |
| Huber  1989(30) | Not specific | 41 | 16 | structural MRI | While the degree of cerebral atrophy was not related to severity of intellectual impairment, patients with severely disturbed intellect exhibited a higher prevalence of skull thickness, focal white matter lesions and anterior temporal lobe abnormalities. |
| Sinforiani  1991(48) | Not specific | 37 | / | structural MRI | Focal white matter lesions were detected in 7 out of 12 subjects. |
| Fiorelli  1992(49) | Not specific | 11 | 14 | FDG/PET | Cortical glucose utilization rate was reduced by about 20% in MD. |
| Chang  1993(50) | Not specific | 22 | 10 | structural MRI  SPECT | Patients with mDM had earlier onset of disease and lower IQs than the pDM patients. The pattern of cerebral perfusion in the mDM patients was consistent with a diffuse brain injury, while cerebral perfusion in pDM showed more minor changes. |
| Censori  1994(51) | Not specific | 25 | 25 | structural MRI | 1. DM patients showed more WMHL than controls, involving all cerebral lobes, particularly at temporal poles; 2. DM patients had significantly more cortical atrophy than controls; 3. No relationship between atrophy and WMHL was found on the MRI scans.; 4. The extent of brain abnormalities (WMHL or atrophy) was not correlated to age, disease duration, physical disability or severity of neuropsychological impairment. |
| Abe  1994(52) | Not specific | 14 | 14 | structural MRI | 1. All patients had ventricular enlargement and white matter abnormalities on MRI; 2. The cognitive severity was variable and there was no difference in neuropsychological testing between patients with mild ventricular dilatation and those with severe dilatation;  3. The white matter abnormalities were the cause of cognitive impairment among patients with DM. |
| Damian  1994(53) | Not specific | 22 | 39 MS | structural MRI | WMLs in DM were symmetrical and mainly subcortical. |
| Damian  1994(54) | Not specific | 28 | / | structural MRI | 1. Disease duration influenced the appearance and amount of white matter lesions on MRI; 2. Quantification of CTG repeat size may allow an early estimate on the probability of brain involvement in DM;  3. Cognitive dysfunction is associated with WML and cerebral atrophy later on in the course. |
| Hashimoto  1995(55) | Not specific | 13 | / | structural MRI | The incidence of a small corpus callosum or ventricular enlargement was higher in CDM than in adult-type DM. |
| Bachmann  1996(56) | Not specific | 40 | / | structural MRI | 1. Cerebral pathology on MRI consisted of diffuse atrophy (68 %), subcortical white matter lesions (65 %), wide Virchow-Robin spaces (38 %) and thickening of the skull (35 %);  2. Cerebral atrophy and extent of white matter disease correlated significantly with mental retardation, duration of disease and CTG fragment amplification. |
| Hund  1997(57) | DM2 | 10 | / | structural MRI | The abnormalities consisted of a homogeneous, symmetric hyperintensity of the periventricular white matter on T2-weighted images. |
| Annane  1998(58) | Not specific | 11 | 11 | PET | The brain metabolism of glucose was impaired in a repeat dependent manner among DM patients. |
| Ogata  1998(59) | Not specific | 12 |  | structural MRI | 1. Pathological findings were severe loss and disordered arrangement of myelin sheaths and axons in addition to heterotopic neurons within anterior temporal white matter； 2. Bilateral ATWML might be a factor for intellectual impairment in DM. |
| Martinello  1999(60) | CDM | 5 | / | structural MRI | CDM patients showed some degree of ventricular dilatation, loosely correlated to the cognitive impairment; in three there was hypoplasia of the corpus callosum and in two mild abnormalities of supratentorial white matter. |
| Meola  1999(61) | DM2 | 20 | 20 | structural MRI H_2_O PET | Impaired visual–spatial function may be present in DM2, and correlates best with a reduction in regional cerebral blood flow observed in H2O PET brain scans rather than with specific structural abnormalities observed on brain MRI. |
| Di Costanzo  2001(62) | Not specific | 41 | 41 | structural MRI | Dilated convexity VRSs might be one of the initial findings in cranial MRI of DM, preceding the appearance of lobar WMLs. |
| Di Costanzo  2001(63) | Not specific | 20 | 20 | structural MRI | 1. All white-matter and occipital and right frontal cortex regions showed a significantly longer T2 in the patients; 2. Only white-matter T2 elongation correlated positively with disease duration. |
| Di Costanzo  2002(64) | DM1 | 5 CDM  and 20 Adult-onset DM1 | / | structural MRI | 1. In CDM1, MRI was characterized by ventriculomegaly and moderate/severe hyperintensity of white matter posterosuperior to trigones, which showed no correlation with the age; 2. MRI in the adult-form dystrophy type 1 was strictly related to disease duration and varied between normal findings, except for temporo-polar white matter lesions, in age-matched patients and ventriculomegaly with white matter hyperintensities in disease duration-matched patients. |
| Di Costanzo  2002(65) | DM1 | 66 | / | structural MRI | Lobar white matter involvement in DM1 seems progressive during the disease and may be characterized initially by large VRSs or mild WMPST hyperintensity, then by small WMLs or moderate WMPST hyperintensity, and finally by more extensive and confluent WMLs or diffuse white matter hyperintensity and by brain atrophy. |
| Kassubek  2003(66) | DM1 and DM2 | 10 DM1 and  9 DM2 | 20 | structural MRI | Global brain atrophy was demonstrated to occur in both DM1 and DM2, but was more severely manifested in DM1 patients; |
| Kornblum  2004(67) | DM1 and DM2 | 10 DM1 and  9 DM2 |  | structural MRI | 1. WML and/or brain atrophy both were found in DM1 and DM2 patients; 2. Anterior temporal WML (ATWML) was exclusively seen in DM1; 3. A high frequency of central nervous system involvement in both disorders. However, temporopolar pathology, previously associated with intellectual dysfunction, seems to be restricted to DM1. |
| Antonini  2004(68) | DM1 | 22 | 22 | structural MRI | 1. Patients with DM1 had significantly reduced brain tissue volumes; 2. Grey matter volume was inversely correlated with age; this inverse the correlation was significantly stronger in DM1 than in controls; 3. Neither the clinical and genetic characteristics nor white matter lesions were correlated with cortical atrophy; 4. Grey matter atrophy predominantly occurred in the bilateral frontal and parietal lobes, as well as in the bilateral middle temporal gyrus, left superior temporal and occipital gyrus. |
| Kuo  2005(69) | DM1 | 2 CDM and 4 Adult-onset DM1 | / | structural MRI | 1. The HWMPST in brain MRI was a distinctive finding observed in congenital DM1, and the severe cognitive impairments were not only attributable to the subcortical WHL;  2. In congenital DM1, the cognitive function exhibited a widespread impairment, distinct from that observed in classic DM1. |
| Vielhaber  2006(70) | DM1 and DM2 | 14 DM1 and 15 DM2 | / | MRS | 1. The concentration of N-acetylaspartate was significantly reduced in all tested brain regions in both DM1 and DM2; 2. In the DM1 patients, a concomitant depletion of creatine and choline levels were found, particularly in the frontal white matter; 3. Although structural abnormalities (cerebral atrophy, WHL) were similar in DM2 and DM1, changes in cerebral metabolites can differentiate between the two disease groups. |
| Ota  2006(71) | Not specific | 11 | 13 | DTI | 1. Significantly lower Fractional anisotropy and higher mean diffusivity values were found in the genu, rostral body, anterior midbody, posterior midbody and splenium in MD patients than in control subjects; 2. Significant negative correlations were observed between the volumes of frontal areas were noted, particularly bilateral motor areas, and the expansion of cytosine thymine guanidine (CTG) triplet. |
| Kuo  2008(72) | DM1 | 7 | / | structural MRI | Subcortical WMLs were correlated with focal dementia in classic DM1 patients. Temporal and insular WMLs may be responsible for the global intellectual dysfunction of adult DM1 patients. |
| Di Costanzo  2008(73) | DM1 | 60 | / | structural MRI | 1. Presence and extent of lobar, temporal or periventricular lesions showed a significant association with the family history of lesions and the disease duration, and no association with the CTG repeat size. 2. Parent-offspring and sibling pairs showed a significant positive concordance for lesion severity. WHLs demonstrate familial aggregation in DM1 and showed no relationship with CTG repeat length. |
| Savio  2011(74) | DM1 | 30 | 30 | structural MRI | The most discriminant voxels were in the caudate nucleus, fronto-parietal lobe and thalamus. |
| Minnerop  2011(75) | DM1 and DM2 | 22 | 22 | structural MRI | Both DM1 and DM2 were serious white matter diseases with prominent callosal body and limbic system affection. WMLs dominated the extent of grey matter changes, which might argue against Wallerian degeneration as the major cause of white matter affection in DM. |
| Magzhanov  2012(76) | DM1 | 20 | 10 | structural MRI | The involvement of the gray matter (cortex atrophy) and the white matter (dilatation of the ventricular system, strengthening of the perivascular spaces, areas of T2 and FLAIR hyperintensity) was observed in the pathological process. |
| Caliandro  2013(77) | DM1 | 29 | 30 | fNIRS | DM1 patients show prefrontal hypometabolism during a specific frontal cognitive task compared to controls. Moreover the rapid temporal discrimination of fNIRS allows revealing the correlation between the PFC hypometabolism and the cognitive performance in DM1 patients. |
| Serra  2014(78) | DM1 | 27 | 16 | fMRI | There is an increase in functional connectivity in the bilateral posterior cingulate and left parietal DMN nodes in DM1 patients compared with controls. Moreover, patients with DM1 showed strong associations between DMN functional connectivity and schizotypal-paranoid traits. |
| Bajrami  2017(79) | DM1 | 13 | / | structural MRI | WMHLs in DM1 patients mainly located in the anterior temporal, frontal, parieto-occipital and periventricular WM regions. |
| Cabada  2017(80) | DM1 | 42 | 42 | DTI | 1. WMLs were significantly more frequent in DM1 patients, and anterior temporal lobe lesions were only observed in the patient; 2. Global and regional cortical volume loss and corpus callosum atrophy were found; 3. Diffuse white matter DTI abnormalities, including fractional anisotropy, mean diffusivity, axial diffusivity, and radial diffusivity were observed with sparing of the internal capsule; 4. Subcortical structures showed volume loss and increased median diffusivity; 5. Only visuospatial impairment was correlated with WMLs and cortical atrophy |
| Park  2018(81) | DM1 | 18 | 20 | DTI  structural MRI | 1. Corticospinal tract involvement reflecting deterioration of the motor tracts may play a significant role in clinical myotonia; 2. A direct relationship was observed between the cortical gray matter volume and DTI measures in the CST. |
| Leddy  2021(82) | DM1 | 29 | 15 | MRI | DM1 presented higher prevalence of anterior temporal lobe lesions, but none in the cerebellum and brainstem. Significantly reduced F values were found within DM1 lesions. |
| Lopez-Titla  2021(83) | DM1 | 22 | 22 | DTI | Despite the pervasive WM integrity loss in DM1 disorder, specific memory impairments were associated to discreet areas of WM deterioration in these patients. |
| Koscik  2021(84) | DM1 | 50 | 69 | DTI | 1. Fractional anisotropy (a measure of WM integrity) throughout the cerebrum was the strongest predictor of grip strength independently of disease duration and genetic burden, while radial diffusivity predicted motor skill (peg board performance). Axial diffusivity did not predict motor outcomes; 2. Tracking changes in WM integrity over time may be a valuable biomarker for tracking therapeutic interventions, such as future gene therapies, for DM1. |
| Cabada  2021(85) | DM1 | 33 | / | DTI and structural MRI | White matter and grey matter involvement in DM1 patients was progressive. Patients experienced a worsening in cognitive impairment that correlates with white matter involvement. |
| Labayru  2022(86) | DM1 | 26 | 57 | DTI | 1. DM1 patients showed a pronounced WM integrity loss over time compared to HC, with a neurodegeneration pattern that suggests a progressive anterior–posterior disconnection; 2. The visuo-construction domain was the most sensitive neuropsychological measure for WM microstructural impairment. |
| Laforce  2022(87) | DM1 | 12 | 2AD | Tau PET | 1. Although DM1 may indeed represent a non-AD Tauopathy, the Tau-PET tracer used in this study was unable to detect an in vivo Tau DM1 signature in this small cohort; 2. Most DM1 participants presented with elevated plasma NfL and GFAP levels |
| Koscik  2023(88) | DM1 | 41 | 69 | DTI | 1. Cerebral white matter integrity and functional outcomes was reduced in DM1; 2. White matter integrity was associated with declining executive function in DM1; 3. Diffusivity in DM1 was associated with reductions in general cognitive ability; 4. Short interval MR-diffusivity may be useful to evaluate DM1 treatment efficacy. |

Note: DM, myotonic dystrophy; DM1, myotonic dystrophy type 1; DM2, myotonic dystrophy type 2; CDM, congenital myotonic dystrophy; ADM, adult-onset myotonic dystrophy; mDM, DM with maternal inheritance; pMD, DM with paternal inheritance; fMRI, functional magnetic resonance imaging; DTI, diffusion tensor imaging; PET, positron emission tomography; SPECT, single photon emission computed tomography; MRS, magnetic resonance spectroscopy; fNIRS, functional near Infrared spectroscopy; ATWML, anterior temporal white matter lesions; WML, white matter lesion; WMHL, white matter hyperintensities lesions; HWMPST, hyperintensity of white matter at the posterior-superior trigone; WM, white matter; HC, healthy control; NFL, Neurofilament Light Chain; GFAP, Glial Fibrillary Acidic Protein; PFC, prefrontal cortex.

**Reference**

1. Rakocevic-Stojanovic V, Peric S, Madzarevic R, Dobricic V, Ralic V, Ilic V, et al. Significant impact of behavioral and cognitive impairment on quality of life in patients with myotonic dystrophy type 1. Clinical neurology and neurosurgery. 2014 Nov;126:76–81.

2. Gaul C, Schmidt T, Windisch G, Wieser T, Müller T, Vielhaber S, et al. Subtle cognitive dysfunction in adult onset myotonic dystrophy type 1 (DM1) and type 2 (DM2). Neurology. 2006 Jul 25;67(2):350–2.

3. Modoni A, Silvestri G, Vita MG, Quaranta D, Tonali PA, Marra C. Cognitive impairment in myotonic dystrophy type 1 (DM1): a longitudinal follow-up study. Journal of neurology. 2008 Nov;255(11):1737–42.

4. Sansone V, Gandossini S, Cotelli M, Calabria M, Zanetti O, Meola G. Cognitive impairment in adult myotonic dystrophies: a longitudinal study. Neurological sciences : official journal of the Italian Neurological Society and of the Italian Society of Clinical Neurophysiology. 2007 Mar;28(1):9–15.

5. Sistiaga A, Urreta I, Jodar M, Cobo AM, Emparanza J, Otaegui D, et al. Cognitive/personality pattern and triplet expansion size in adult myotonic dystrophy type 1 (DM1): CTG repeats, cognition and personality in DM1. Psychol Med. 2010 Mar;40(3):487–95.

6. Angeard N, Jacquette A, Gargiulo M, Radvanyi H, Moutier S, Eymard B, et al. A new window on neurocognitive dysfunction in the childhood form of myotonic dystrophy type 1 (DM1). Neuromuscul Disord. 2011 Jul;21(7):468–76.

7. Baldanzi S, Bevilacqua F, Lorio R, Volpi L, Simoncini C, Petrucci A, et al. Disease awareness in myotonic dystrophy type 1: an observational cross-sectional study. Orphanet journal of rare diseases. 2016 Apr 4;11:34.

8. Fujino H, Shingaki H, Suwazono S, Ueda Y, Wada C, Nakayama T, et al. Cognitive impairment and quality of life in patients with myotonic dystrophy type 1. Muscle & nerve. 2018 May;57(5):742–8.

9. Peric S, Brajkovic L, Belanovic B, Ilic V, Salak-Djokic B, Basta I, et al. Brain positron emission tomography in patients with myotonic dystrophy type 1 and type 2. Journal of the Neurological Sciences. 2017 Jul;378:187–92.

10. Gallais B, Gagnon C, Mathieu J, Richer L. Cognitive decline over time in adults with myotonic dystrophy type 1: A 9-year longitudinal study. Neuromuscular disorders : NMD. 2017 Jan;27(1):61–72.

11. Peric S, Gunjic I, Delic N, Stojiljkovic Tamas O, Salak-Djokic B, Pesovic J, et al. Cognitive assessment in patients with myotonic dystrophy type 2. Neuromuscular disorders : NMD. 2022 Sep;32(9):743–8.

12. Zalonis I, Bonakis A, Christidi F, Vagiakis E, Papageorgiou SG, Kalfakis N, et al. Toward understanding cognitive impairment in patients with myotonic dystrophy type 1. Archives of clinical neuropsychology : the official journal of the National Academy of Neuropsychologists. 2010 Jun;25(4):303–13.

13. Douniol M, Jacquette A, Cohen D, Bodeau N, Rachidi L, Angeard N, et al. Psychiatric and cognitive phenotype of childhood myotonic dystrophy type 1. Dev Med Child Neurol. 2012 Oct;54(10):905–11.

14. Labayru G, Aliri J, Zulaica M, López de Munain A, Sistiaga A. Age-related cognitive decline in myotonic dystrophy type 1: An 11-year longitudinal follow-up study. Journal of neuropsychology. 2020 Mar;14(1):121–34.

15. Sweere DJJ, Moelands SVL, Klinkenberg S, Leenen L, Hendriksen JGM, Braakman HMH. Cognitive phenotype of childhood myotonic dystrophy type 1: A multicenter pooled analysis. Muscle & nerve. 2023 Jul;68(1):57–64.

16. Kleberg JL, Lindberg C, Winblad S. Facial memory deficits in myotonic dystrophy type 1. Acta Neurol Scand. 2014 Nov;130(5):312–8.

17. Woo J, Lee HW, Park JS. Differences in the pattern of cognitive impairments between juvenile and adult onset myotonic dystrophy type 1. Journal of clinical neuroscience : official journal of the Neurosurgical Society of Australasia. 2019 Oct;68:92–6.

18. Rubinsztein JS, Rubinsztein DC, McKenna PJ, Goodburn S, Holland AJ. Mild myotonic dystrophy is associated with memory impairment in the context of normal general intelligence. J Med Genet. 1997 Mar;34(3):229–33.

19. Ekström AB, Hakenäs-Plate L, Tulinius M, Wentz E. Cognition and adaptive skills in myotonic dystrophy type 1: a study of 55 individuals with congenital and childhood forms. Dev Med Child Neurol. 2009 Dec;51(12):982–90.

20. Filli L, Schwegler S, Meyer C, Killeen T, Easthope CS, Broicher SD, et al. Characterizing cognitive-motor impairments in patients with myotonic dystrophy type 1. Neuromuscular disorders : NMD. 2020 Jun;30(6):510–20.

21. Kobayakawa M, Tsuruya N, Kawamura M. Theory of mind impairment in adult-onset myotonic dystrophy type 1. Neuroscience research. 2012 Apr;72(4):341–6.

22. Modoni A, Silvestri G, Pomponi MG, Mangiola F, Tonali PA, Marra C. Characterization of the pattern of cognitive impairment in myotonic dystrophy type 1. Archives of neurology. 2004 Dec;61(12):1943–7.

23. Ricci FS, Vacchetti M, Brusa C, D’Alessandro R, La Rosa P, Martone G, et al. Cognitive, neuropsychological and emotional-behavioural functioning in a sample of children with myotonic dystrophy type 1. European journal of paediatric neurology : EJPN : official journal of the European Paediatric Neurology Society. 2022 Jul;39:59–64.

24. Woodward JB, Heaton RK, Simon DB, Ringel SP. Neuropsychological findings in myotonic dystrophy. Journal of clinical neuropsychology. 1982 Dec;4(4):335–42.

25. Steyaert J, Umans S, Willekens D, Legius E, Pijkels E, De Die‐Smulders C, et al. A study of the cognitive and psychological profile in 16 children with congenital or juvenile myotonic dystrophy. Clinical Genetics. 1997 Sep;52(3):135–41.

26. Romeo V, Pegoraro E, Ferrati C, Squarzanti F, Sorarù G, Palmieri A, et al. Brain involvement in myotonic dystrophies: neuroimaging and neuropsychological comparative study in DM1 and DM2. J Neurol. 2010 Aug;257(8):1246–55.

27. Fortin J, Côté I, Gagnon C, Gallais B. Do classical and computerized cognitive tests have equal intrarater reliability in myotonic dystrophy type 1? Neuromuscular disorders : NMD. 2023 Jun;33(6):490–7.

28. Tuikka RA, Laaksonen RK, Somer HV. Cognitive function in myotonic dystrophy: a follow-up study. European neurology. 1993;33(6):436–41.

29. Bird TD, Follett C, Griep E. Cognitive and personality function in myotonic muscular dystrophy. J Neurol Neurosurg Psychiatry. 1983 Nov;46(11):971–80.

30. Huber SJ, Kissel JT, Shuttleworth EC, Chakeres DW, Clapp LE, Brogan MA. Magnetic resonance imaging and clinical correlates of intellectual impairment in myotonic dystrophy. Arch Neurol. 1989 May;46(5):536–40.

31. Palmer BW, Boone KB, Chang L, Lee A, Black S. Cognitive deficits and personality patterns in maternally versus paternally inherited myotonic dystrophy. J Clin Exp Neuropsychol. 1994 Oct;16(5):784–95.

32. Winblad S, Samuelsson L, Lindberg C, Meola G. Cognition in myotonic dystrophy type 1: a 5-year follow-up study. European journal of neurology. 2016 Sep;23(9):1471–6.

33. Winblad S, Lindberg C, Hansen S. Cognitive deficits and CTG repeat expansion size in classical myotonic dystrophy type 1 (DM1). Behav Brain Funct. 2006 May 15;2:16.

34. Tremblay M, Muslemani S, Côté I, Gagnon C, Fortin J, Gallais B. Accomplishment of instrumental activities of daily living and its relationship with cognitive functions in adults with myotonic dystrophy type 1 childhood phenotype: an exploratory study. BMC psychology. 2021 Apr 17;9(1):56.

35. Díaz-Leiva J, Cabada-Giadás T, Seijas-Gómez R, Jericó-Pascual I, López-Sala P, Iridoy-Zulet M. Neuropsychological profile in patients with myotonic dystrophy type 1: a four-year follow-up study. Revista de neurologia. 2020 Jun 1;70(11):406–12.

36. Fujino H, Suwazono S, Ueda Y, Kobayashi M, Nakayama T, Imura O, et al. Longitudinal Changes in Neuropsychological Functioning in Japanese Patients with Myotonic Dystrophy Type 1: A Five Year Follow-Up Study. Journal of neuromuscular diseases. 2023;10(6):1083–92.

37. Gliem C, Minnerop M, Roeske S, Gärtner H, Schoene-Bake JC, Adler S, et al. Tracking the brain in myotonic dystrophies: A 5-year longitudinal follow-up study. PLoS One. 2019;14(3):e0213381.

38. Labayru G, Diez I, Sepulcre J, Fernández E, Zulaica M, Cortés JM, et al. Regional brain atrophy in gray and white matter is associated with cognitive impairment in Myotonic Dystrophy type 1. NeuroImage Clinical. 2019;24:102078.

39. Meola G, Sansone V, Perani D, Scarone S, Cappa S, Dragoni C, et al. Executive dysfunction and avoidant personality trait in myotonic dystrophy type 1 (DM-1) and in proximal myotonic myopathy (PROMM/DM-2). Neuromuscular disorders : NMD. 2003 Dec;13(10):813–21.

40. Schneider-Gold C, Bellenberg B, Prehn C, Krogias C, Schneider R, Klein J, et al. Cortical and Subcortical Grey and White Matter Atrophy in Myotonic Dystrophies Type 1 and 2 Is Associated with Cognitive Impairment, Depression and Daytime Sleepiness. PloS one. 2015;10(6):e0130352.

41. Theodosiou T, Christidi F, Xirou S, Karavasilis E, Bede P, Papadopoulos C, et al. Executive Dysfunction, Social Cognition Impairment, and Gray Matter Pathology in Myotonic Dystrophy Type 2: A Pilot Study. Cognitive and behavioral neurology : official journal of the Society for Behavioral and Cognitive Neurology. 2022 Sep 1;35(3):204–11.

42. Langbehn KE, van der Plas E, Moser DJ, Long JD, Gutmann L, Nopoulos PC. Cognitive function and its relationship with brain structure in myotonic dystrophy type 1. Journal of neuroscience research. 2021 Jan;99(1):190–9.

43. Caso F, Agosta F, Peric S, Rakočević-Stojanović V, Copetti M, Kostic VS, et al. Cognitive impairment in myotonic dystrophy type 1 is associated with white matter damage. PloS one. 2014;9(8):e104697.

44. Baldanzi S, Cecchi P, Fabbri S, Pesaresi I, Simoncini C, Angelini C, et al. Relationship between neuropsychological impairment and grey and white matter changes in adult-onset myotonic dystrophy type 1. NeuroImage Clinical. 2016;12:190–7.

45. Serra L, Scocchia M, Meola G, D’Amelio M, Bruschini M, Silvestri G, et al. Ventral tegmental area dysfunction affects decision-making in patients with myotonic dystrophy type-1. Cortex; a journal devoted to the study of the nervous system and behavior. 2020 Jul;128:192–202.

46. Van Spaendonck KP, Ter Bruggen JP, Weyn Banningh EW, Maassen BA, Van de Biezenbos JB, Gabreëls FJ. Cognitive function in early adult and adult onset myotonic dystrophy. Acta Neurol Scand. 1995 Jun;91(6):456–61.

47. Glantz RH, Wright RB, Huckman MS, Garron DC, Siegel IM. Central nervous system magnetic resonance imaging findings in myotonic dystrophy. Arch Neurol. 1988 Jan;45(1):36–7.

48. Sinforiani E, Sandrini G, Martelli A, Mauri M, Uggetti C, Bono G, et al. Cognitive and neuroradiological findings in myotonic dystrophy. Funct Neurol. 1991;6(4):377–84.

49. Fiorelli M, Duboc D, Mazoyer BM, Blin J, Eymard B, Fardeau M, et al. Decreased cerebral glucose utilization in myotonic dystrophy. Neurology. 1992 Jan;42(1):91–4.

50. Chang L, Anderson T, Migneco OA, Boone K, Mehringer CM, Villanueva-Meyer J, et al. Cerebral abnormalities in myotonic dystrophy. Cerebral blood flow, magnetic resonance imaging, and neuropsychological tests. Arch Neurol. 1993 Sep;50(9):917–23.

51. Censori B, Provinciali L, Danni M, Chiaramoni L, Maricotti M, Foschi N, et al. Brain involvement in myotonic dystrophy: MRI features and their relationship to clinical and cognitive conditions. Acta neurologica Scandinavica. 1994 Sep;90(3):211–7.

52. Abe K, Fujimura H, Toyooka K, Yorifuji S, Nishikawa Y, Hazama T, et al. Involvement of the central nervous system in myotonic dystrophy. Journal of the Neurological Sciences. 1994 Dec;127(2):179–85.

53. Damian MS, Schilling G, Bachmann G, Simon C, Stöppler S, Dorndorf W. White matter lesions and cognitive deficits: relevance of lesion pattern? Acta Neurol Scand. 1994 Dec;90(6):430–6.

54. Damian MS, Bachmann G, Koch MC, Schilling G, Stöppler S, Dorndorf W. Brain disease and molecular analysis in myotonic dystrophy. Neuroreport. 1994 Dec 20;5(18):2549–52.

55. Hashimoto T, Tayama M, Miyazaki M, Murakawa K, Kawai H, Nishitani H, et al. Neuroimaging study of myotonic dystrophy. I. Magnetic resonance imaging of the brain. Brain Dev. 1995;17(1):24–7.

56. Bachmann G, Damian MS, Koch M, Schilling G, Fach B, Stöppler S. The clinical and genetic correlates of MRI findings in myotonic dystrophy. Neuroradiology. 1996 Oct;38(7):629–35.

57. Hund E, Jansen O, Koch MC, Ricker K, Fogel W, Niedermaier N, et al. Proximal myotonic myopathy with MRI white matter abnormalities of the brain. Neurology. 1997 Jan;48(1):33–7.

58. Annane D, Fiorelli M, Mazoyer B, Pappata S, Eymard B, Radvanyi H, et al. Impaired cerebral glucose metabolism in myotonic dystrophy: a triplet-size dependent phenomenon. Neuromuscular Disorders. 1998 Feb;8(1):39–45.

59. Ogata A, Terae S, Fujita M, Tashiro K. Anterior temporal white matter lesions in myotonic dystrophy with intellectual impairment: an MRI and neuropathological study. Neuroradiology. 1998 Jul 22;40(7):411–5.

60. Martinello F, Piazza A, Pastorello E, Angelini C, Trevisan CP. Clinical and neuroimaging study of central nervous system in congenital myotonic dystrophy. Journal of neurology. 1999 Mar;246(3):186–92.

61. Meola G, Sansone V, Perani D, Colleluori A, Cappa S, Cotelli M, et al. Reduced cerebral blood flow and impaired visual-spatial function in proximal myotonic myopathy. Neurology. 1999 Sep 22;53(5):1042–50.

62. Di Costanzo A, Di Salle F, Santoro L, Bonavita V, Tedeschi G. Dilated Virchow-Robin spaces in myotonic dystrophy: frequency, extent and significance. Eur Neurol. 2001;46(3):131–9.

63. Di Costanzo A, Di Salle F, Santoro L, Bonavita V, Tedeschi G. T2 relaxometry of brain in myotonic dystrophy. Neuroradiology. 2001 Mar;43(3):198–204.

64. Di Costanzo A, Di Salle F, Santoro L, Bonavita V, Tedeschi G. Brain MRI features of congenital- and adult-form myotonic dystrophy type 1: case-control study. Neuromuscular Disorders. 2002 Jun;12(5):476–83.

65. Di Costanzo A, Di Salle F, Santoro L, Tessitore A, Bonavita V, Tedeschi G. Pattern and significance of white matter abnormalities in myotonic dystrophy type 1: an MRI study. J Neurol. 2002 Sep;249(9):1175–82.

66. Kassubek J, Juengling FD, Hoffmann S, Rosenbohm A, Kurt A, Jurkat-Rott K, et al. Quantification of brain atrophy in patients with myotonic dystrophy and proximal myotonic myopathy: a controlled 3-dimensional magnetic resonance imaging study. Neuroscience letters. 2003 Sep 11;348(2):73–6.

67. Kornblum C, Reul J, Kress W, Grothe C, Amanatidis N, Klockgether T, et al. Cranial magnetic resonance imaging in genetically proven myotonic dystrophy type 1 and 2. J Neurol [Internet]. 2004 Jun [cited 2023 Dec 5];251(6). Available from: http://link.springer.com/10.1007/s00415-004-0408-1

68. Antonini G, Mainero C, Romano A, Giubilei F, Ceschin V, Gragnani F, et al. Cerebral atrophy in myotonic dystrophy: a voxel based morphometric study. J Neurol Neurosurg Psychiatry. 2004 Nov;75(11):1611–3.

69. Kuo HC, Hsiao KM, Chen CJ, Hsieh YC, Huang CC. Brain magnetic resonance image changes in a family with congenital and classic myotonic dystrophy. Brain & development. 2005 Jun;27(4):291–6.

70. Vielhaber S, Jakubiczka S, Gaul C, Schoenfeld MA, Debska-Vielhaber G, Zierz S, et al. Brain 1H magnetic resonance spectroscopic differences in myotonic dystrophy type 2 and type 1. Muscle Nerve. 2006 Aug;34(2):145–52.

71. Ota M, Sato N, Ohya Y, Aoki Y, Mizukami K, Mori T, et al. Relationship between diffusion tensor imaging and brain morphology in patients with myotonic dystrophy. Neurosci Lett. 2006 Oct 30;407(3):234–9.

72. Kuo HC, Hsieh YC, Wang HM, Chuang WL, Huang CC. Correlation among subcortical white matter lesions, intelligence and CTG repeat expansion in classic myotonic dystrophy type 1. Acta Neurol Scand. 2008 Feb;117(2):101–7.

73. Di Costanzo A, Santoro L, de Cristofaro M, Manganelli F, Di Salle F, Tedeschi G. Familial aggregation of white matter lesions in myotonic dystrophy type 1. Neuromuscul Disord. 2008 Apr;18(4):299–305.

74. Savio A, García-Sebastián MT, Chyzyk D, Hernandez C, Graña M, Sistiaga A, et al. Neurocognitive disorder detection based on feature vectors extracted from VBM analysis of structural MRI. Computers in biology and medicine. 2011 Aug;41(8):600–10.

75. Minnerop M, Weber B, Schoene-Bake JC, Roeske S, Mirbach S, Anspach C, et al. The brain in myotonic dystrophy 1 and 2: evidence for a predominant white matter disease. Brain. 2011 Dec;134(Pt 12):3530–46.

76. Magzhanov RV, Saĭfullina EV, Mukhametova RR, Mukhamedrakhimov RR. Cognitive disorders in patients with myotonic dystrophy type I: a clinical and magnetic resonance study. Zhurnal nevrologii i psikhiatrii imeni SS Korsakova. 2012;112(4):18–22.

77. Caliandro P, Silvestri G, Padua L, Bianchi ML, Simbolotti C, Russo G, et al. fNIRS evaluation during a phonemic verbal task reveals prefrontal hypometabolism in patients affected by myotonic dystrophy type 1. Clinical neurophysiology : official journal of the International Federation of Clinical Neurophysiology. 2013 Nov;124(11):2269–76.

78. Serra L, Silvestri G, Petrucci A, Basile B, Masciullo M, Makovac E, et al. Abnormal functional brain connectivity and personality traits in myotonic dystrophy type 1. JAMA Neurol. 2014 May;71(5):603–11.

79. Bajrami A, Azman F, Yayla V, Cagirici S, Keskinkiliç C, Sozer N. MRI findings and cognitive functions in a small cohort of myotonic dystrophy type 1: Retrospective analyses. Neuroradiol J. 2017 Feb;30(1):23–7.

80. Cabada T, Iridoy M, Jericó I, Lecumberri P, Seijas R, Gargallo A, et al. Brain Involvement in Myotonic Dystrophy Type 1: A Morphometric and Diffusion Tensor Imaging Study with Neuropsychological Correlation. Arch Clin Neuropsychol. 2017 Jun 1;32(4):401–12.

81. Park JS, Song H, Jang KE, Cha H, Lee SH, Hwang SK, et al. Diffusion tensor imaging and voxel-based morphometry reveal corticospinal tract involvement in the motor dysfunction of adult-onset myotonic dystrophy type 1. Sci Rep. 2018 Oct 22;8(1):15592.

82. Leddy S, Serra L, Esposito D, Vizzotto C, Giulietti G, Silvestri G, et al. Lesion distribution and substrate of white matter damage in myotonic dystrophy type 1: Comparison with multiple sclerosis. NeuroImage Clinical. 2021;29:102562.

83. Lopez-Titla MM, Chirino A, Cruz Solis SV, Hernandez-Castillo CR, Diaz R, Márquez-Quiroz LDC, et al. Cognitive Decline and White Matter Integrity Degradation in Myotonic Dystrophy Type I. Journal of neuroimaging : official journal of the American Society of Neuroimaging. 2021 Jan;31(1):192–8.

84. Koscik TR, Van Der Plas E, Gutmann L, Cumming SA, Monckton DG, Magnotta V, et al. White matter microstructure relates to motor outcomes in myotonic dystrophy type 1 independently of disease duration and genetic burden. Sci Rep. 2021 Mar 1;11(1):4886.

85. Cabada T, Díaz J, Iridoy M, López P, Jericó I, Lecumberri P, et al. Longitudinal study in patients with myotonic dystrophy type 1: correlation of brain MRI abnormalities with cognitive performances. Neuroradiology. 2021 Jul;63(7):1019–29.

86. Labayru G, Camino B, Jimenez-Marin A, Garmendia J, Villanua J, Zulaica M, et al. White matter integrity changes and neurocognitive functioning in adult-late onset DM1: a follow-up DTI study. Sci Rep. 2022 Mar 7;12(1):3988.

87. Laforce RJ, Dallaire-Théroux C, Racine AM, Dent G, Salinas-Valenzuela C, Poulin E, et al. Tau positron emission tomography, cerebrospinal fluid and plasma biomarkers of neurodegeneration, and neurocognitive testing: an exploratory study of participants with myotonic dystrophy type 1. Journal of neurology. 2022 Jul;269(7):3579–87.

88. Koscik TR, Van Der Plas E, Long JD, Cross S, Gutmann L, Cumming SA, et al. Longitudinal changes in white matter as measured with diffusion tensor imaging in adult-onset myotonic dystrophy type 1. Neuromuscular Disorders. 2023 Aug;33(8):660–9.
